# Epistasis between *Pax6*^*Sey*^ and genetic background reinforces the value of defined hybrid mouse models for therapeutic trials

**DOI:** 10.1038/s41434-018-0043-6

**Published:** 2018-09-26

**Authors:** Jack W. Hickmott, Uvini Gunawardane, Kimberly Jensen, Andrea J. Korecki, Elizabeth M. Simpson

**Affiliations:** 1 Centre for Molecular Medicine and Therapeutics at BC Children’s Hospital, University of British Columbia, Vancouver, BC Canada; 2 Department of Medical Genetics, University of British Columbia, Vancouver, BC Canada; 3 Department of Psychiatry, University of British Columbia, Vancouver, BC Canada; 4 Department of Ophthalmology and Visual Science, University of British Columbia, Vancouver, BC Canada

## Abstract

The small eye (*Sey*) mouse is a model of PAX6-aniridia syndrome (aniridia). Aniridia, a congenital ocular disorder caused by heterozygous loss-of-function mutations in *PAX6*, needs new vision saving therapies. However, high phenotypic variability in *Sey* mice makes development of such therapies challenging. We hypothesize that genetic background is a major source of undesirable variability in *Sey* mice. Here we performed a systematic quantitative examination of anatomical, histological, and molecular phenotypes on the inbred C57BL/6J, hybrid B6129F1, and inbred 129S1/SvImJ backgrounds. The *Sey* allele significantly reduced eye weight, corneal thickness, PAX6 mRNA and protein levels, and elevated blood glucose levels. Surprisingly, *Pax6*^*Sey/Sey*^ brains had significantly elevated *Pax6* transcripts compared to *Pax6*^*+/+*^ embryos. Genetic background significantly influenced 12/24 measurements, with inbred strains introducing severe ocular and blood sugar phenotypes not observed in hybrid mice. Additionally, significant interactions (epistasis) between *Pax6* genotype and genetic background were detected in measurements of eye weight, cornea epithelial thickness and cell count, retinal mRNA levels, and blood glucose levels. The number of epistatic interactions was reduced in hybrid mice. In conclusion, severe phenotypes in the unnatural inbred strains reinforce the value of more naturalistic F1 hybrid mice for the development of therapies for aniridia and other disorders.

## Introduction

In humans, aniridia is a penetrant monogenic disorder with high phenotypic variability, even between family members with the same mutation [1]. Loss-of-function paired box 6 (*PAX6*) mutations, most frequently premature termination codons (http://lsdb.hgu.mrc.ac.uk/home.php?select_db=PAX6) [2], cause *PAX6*-aniridia syndrome (aniridia, OMIM: 106210). The result of PAX6 haploinsufficiency, aniridia is a rare genetic disorder predominantly affecting the eyes, central nervous system, and pancreas [3–13]. People with aniridia are born with low vision, with diagnosis occurring shortly thereafter when the eponymous iris hypoplasia is readily detectable. In addition to congenital fovea hypoplasia and lens abnormalities that often reduce vision from birth, cataracts, glaucoma, corneal keratopathy, and pannus can progressively obscure vision. The consequence of such progressive visual impairments is often blindness in young adulthood [14, 15], necessitating the development of new vision saving therapies. While environmental factors are likely to contribute to the reported variability, epistasis, the non-additive interaction between genetic loci, may also be a factor influencing how the disorder presents, and how it should be treated [16, 17].

The small eye (*Sey*) mouse has a premature termination codon in *Pax6*, mimicking the most frequent aniridia causing mutations. Like human *PAX6* mutations, *Sey* is penetrant, producing a phenotype that mirrors human aniridia: iris hypoplasia, lens abnormalities, corneal keratopathy, and pannus [4, 18–29]. However, the phenotype is highly variable, even between genetically identical mice [14, 21, 26, 30, 31]. While mimicking the variability of the human disease state is desirable, variability beyond that seen in humans has also been reported, and may confound the development of new therapeutics [30]. Therefore, identifying and limiting these undesirable sources of variability can help accelerate the development of new treatments for aniridia.

Variability in mouse studies is increased by a myriad of sources. For instance, different mutant alleles of the same gene can impact how a phenotype presents [32]. Similarly, genetic background (bkgd) is an important consideration. Inbred mice are a useful tool, but genetically they deviate considerably from humans, as they are homozygous at all loci, lacking in the diversity found in freely reproducing environments [33–35]. This creates an unnatural situation making some phenotypes frail in a way that does not reflect the biology under study and introduces extraneous variables [30, 36]. Finally, environmental variables such as cage conditions, handling practices, and even the experimenter can increase variability [37–39]. Studies of the *Sey* allele have been conducted on numerous different bkgds and at various timepoints, often focusing on embryonic development. Consequently, before developing new therapeutics for aniridia, it would be beneficial to generate benchmark measurements of the *Sey* phenotype, at a later timepoint more relevant to the treatment of human aniridia. Additionally, it is important that we identify sources of unnatural variation in model organisms, so that new therapies are built around the appropriate underlying biology, not artifacts of the laboratory environment. This helps ensure that outcome measures are relevant to the clinical features targeted for therapy, and minimizes unnecessary variability, which can drive up costs while diminishing the reliability of the findings. Here we pursued the hypothesis that bkgd is a major source of undesirable variability in *Sey* mice.

In this work we investigate how bkgd can introduce variability in the *Sey* mouse, and the underlying mechanism responsible, by conducting a quantitative analysis of the *Pax6*^*Sey/+*^ (heterozygous (Het)) and *Pax6*^*Sey/Sey*^ (homozygous (Hom)) phenotypes on three bkgds: C57BL/6J (B6), B6129F1 (F1), and 129S1/SvImJ (129). B6 was selected as C57BL mice are the most commonly studied inbred mouse strain [40–42], serving in numerous *Pax6* studies [23, 24, 43–46]. F1 mice were studied as they are the genetically defined hybrid of B6 and 129 and are often used [34, 47, 48]. 129 was selected as they have been the most commonly used strain for genetic targeting in embryonic stem cells [34, 42]. Rather than searching for modifier genes, we instead looked broadly at how bkgd influences the *Sey* phenotype to address the question: which bkgd should be used for developing new therapies. Examining the eye, brain, and pancreas, we measured and compared anatomical, histological, and molecular aspects of the *Sey* phenotype, and the relationship between *Pax6* genotypes and bkgds. Surprisingly, we found that choice of bkgd can introduce significant variability. We also revealed epistatic interactions between genotype and bkgd, where the resulting phenotype differs from that expected, such that the combination creates a unique or exaggerated phenotype. Using this data, we conclude that the B6129F1 hybrid bkgd is a better choice for therapeutic development than the commonly used inbred strains.

## Methods

### Mouse husbandry

All mice were bred and maintained in the pathogen-free mouse core facility of the Center for Molecular Medicine and Therapeutics at The University of British Columbia. Animal work was performed in accordance with the guidelines set by the Canadian Council on Animal Care (CCAC) and adhered to the ARVO statement for the use of animals in ophthalmic and vision research. The *Sey* mutation was maintained on inbred C57BL/6J (The Jackson Laboratory (JAX), Stock #000664, Bar Harbor, ME) and 129S1/SvImJ (JAX, Stock #002448) strains of mice backcrossed at least 10 generations. B6129F1 mice were generated by crossing B6 dams to 129 sires to produce F1 progeny. Mice were kept on a 7 am–8 pm light cycle and had food and water *ad libitum* unless stated otherwise.

Adult mice, ages three to six months, were sacrificed by cervical dislocation, imaged under a Leica MZ125 microscope with a CoolSnap-Procf camera (Leica Microsystems, Wetzlar, DE), and the eyes were immediately enucleated. Eyes used for histological examination were prepared as previously described [49]. Briefly, they were enucleated and incubated in 4% PFA at 4^o^C for 2 h, weighed, transferred to 25% sucrose, and stored at 4^o^C until used. Eyes used for molecular characterization were placed in room temperature PBS, the cornea and retina were dissected under a dissecting microscope, placed in separate Nunc Cryotubes (MilliporeSigma, St. Louis, MI, Catalog #V7634), flash frozen in liquid nitrogen, and stored at −80^o^C.

Embryonic day 18.5 (E18.5) fetuses were generated using a previously described timed pregnancy protocol [50]. Het dams and sires were bred, producing *Pax6*^*+/+*^ (wild type (Wt)), Het, and Hom offspring. On E18.5, pregnant dams were fasted for 2 h, and then sacrificed by cervical dislocation followed by decapitation. Embryos were photographed and then sacrificed by decapitation. Blood glucose readings were immediately taken from sacrificed fetuses, and then brains were dissected and divided along the longitudinal fissure. Each hemisphere was placed into separate Nunc Cryotubes, flash frozen in liquid nitrogen, and stored at −80^o^C. Additionally, tail tip samples were taken for genotyping by PCR.

### *Pax6* genotyping and RT-ddPCR

PCR was performed on genomic DNA from the digested tail tips of E18.5 embryos, and ear notches from adult mice. As there is no literature describing the influence of parent of origin or imprinting on *Pax6*, all Het mice for RNA analysis were bred such that the *Sey* allele came from the sire. Primers were designed to bind to the *Sey* (oEMS6073: CTGAGCTTCATCCGAGTCTTCTTA) and Wt alleles (oEMS6076: AACACCAACTCCATCAGTTCTAATG) on exon 8 of *Pax6*.

RNA was isolated using the QIAGEN AllPrep kit (QIAGEN, Hilden, DE, Catalog #80204), following the provided protocol. RNA integrity and quantity were assessed by the CMMT core sequencing service on Agilent 2100 bioanalyzer RNA 6000 Nano chips (Agilent Technologies, Santa Clara, CA). Reverse transcription was performed using the SuperScript™ VILO™ cDNA Synthesis Kit and Master Mix (Thermo Fisher Scientific, Waltham, MA, Catalog # 11754050) as directed. For reverse transcriptase Droplet Digital PCR (RT-ddPCR) cDNA was diluted to a standard concentration of 0.1 ng and tested on a QX100 Droplet Digital PCR system (Bio Rad, Hercules, CA) as directed. Custom Taqman primers with specificity to either the *Sey* (*Sey* assay) or wild-type (Wt assay) allele were designed. For both assays the forward primer binds to exon 7 (oEMS6130: ACTTCAGTACCAGGGCAAC) and the probe binds to exon 8 (oEMS6131: AACTGATGGAGTTGGTGTTCTCTCCC). The reverse primers for the *Sey* (oEMS6132: GAGCTTCATCCGAGTCTTCTTA) and Wt (oEMS6133: GAGCTTCATCCGAGTCTTCTTC) assays bind to exon 8, with the 3ʹ end complementary to the *Sey* or Wt allele, respectively, with the exception of a mismatch at the 3ʹ penultimate nucleotide, which was intentionally introduced to improve the specificity of the assay as previously described [51]. *Pax6* transcript measurements were normalized to the geometric mean of three housekeeping genes using commercially available assays: *Pgk1*, *B2m*, and *Tfrc* (Integrated DNA Technologies, Coralville, IO, Assay# Mm.PT.39a.22214854, Mm.PT.58.10497647, and Mm.PT.58.9333140, respectively) [52].

### Western blotting

As there is no literature describing the influence of parent of origin or imprinting on *Pax6*, all Het mice for protein analysis were bred such that the *Sey* allele came from the sire. Proteins were extracted by homogenizing tissue for 30 s in radioimmunoprecipitation assay buffer (150 mM sodium chloride, 1.0% sodium deoxycholate, 1.0% nonyl phenoxypolyethoxylethanol, 0.1% sodium dodecyl sulfate, & 25 mM Tris pH 7.8) containing 1% cOmplete™ protease inhibitor (Roche Diagnostics GmbH, Mannheim, DE, Catalog # 11 697 498 001), 1% phenylmethylsulfonyl fluoride (MilliporeSigma, Catalog # P7626), and 1% sodium orthovanadate (MilliporeSigma, Catalog # P6508). Homogenates were sonicated 2 × for 10 s, rotated for 15 min at 40 °C, and spun down. Supernatant was transferred to a fresh tube and stored at − 80°C. Protein samples were quantified using a DC Protein kit (Bio Rad, Catalog# 500–0113, 500–0114, 500–0015) as directed. For E18.5 brains, adult retinas, and adult corneas, 29, 6.5, and 3.4 µg of protein, respectively, were assessed by western blot using the NuPAGE gel and buffer system (Thermo Fisher Scientific, Catalog# NP0341BOX, NP0002, NP0005, NP0006-1, NP0008, & NP0009), Immun-Blot PVDF Membranes (Bio Rad; Catalog # 162–0255), and the XCell II Blot Module (Thermo Fisher Scientific, Catalog # EI9051) as directed. Membranes were blocked for 1 h in 5% skim milk (Becton, Dickson, and company, Franklin Lakes, NJ, Catalog # DF0032173) labeled with antibodies against PAX6 (7.5:10,000; Biolegend, San Diego, CA, Catalog # 901301) and Lamin B1 (7.5:10,000; Santa Cruz Biotechnology, Dallas, TX, Catalog # sc-30264) overnight at 4^o^C, labeled with secondary antibodies against Rabbit (1:5000; Thermo Fisher Scientific, Catalog # A10043) and Goat (1:5000; Rockland, Limerick, PA, Catalog # 605-731-002) and imaged on a LiCOR Odyssey (Li-COR Biosciences, Lincoln, NE). PAX6 measurements were standardized to Lamin β1 measurements. Lanes where the blotting was weak, with Lamin β1 below 2000 arbitrary units, were excluded from analysis.

### Histological analysis

As there is no literature describing the influence of parent of origin or imprinting on *Pax6*, Het mice for histological analysis were bred such that the *Sey* allele was inherited from either the sire or the dam. As expected, our data showed no influence of parent of origin by one-way analysis of variance (ANOVA). Eyes were embedded, sectioned, and stained with Hoechst as previously described [50]. Imaging was performed on a Leica SP8 Confocal microscope (Leica Microsystems) at 40x magnification and raw image files were converted to composite tagged image file format (TIFF) files using ImageJ software (http://imagej.nih.gov/ij/, version 1.48) with the Bio-Formats plugin (http://www.openmicroscopy.org/site/support/bio-formats5.1/users/imagej/). Tiling images of whole eyes were taken using a Bx61 fluorescent microscope (Olympus Corporation, Tokyo, JP) and saved as TIFFs using ImageJ.

Cell counting and thickness measurements were done blinded to genetic background and *Pax6* genotype. For each eye, three non-overlapping images were taken for analysis, all within 500 µm of the optic nerve for the retina, or above the pupil for the cornea. In the cornea, structural abnormalities such as keratolenticular adhesions were excluded from measurements. Counts were performed manually using the cell counter tool in ImageJ. All cells in the ganglion cell layer were counted, while the number of cells in the inner and outer nuclear layers was calculated by counting one vertical row of cells in the center of each window, and one horizontal row of cells along the edge of the layer. The vertical and horizontal counts were then multiplied together to give an estimate of the cell number, per layer, in a 200 µm window of the retina. Measurements and counts from three images were averaged to produce final figures for each animal. Four different animals were used for each genotype and bkgd. Images of embryonic eyes were examined blinded to genotype and bkgd. For measurements of embryonic whole eye area and non-pigmented area, both were manually circled, and the corresponding areas were calculated using ImageJ.

### Statistical analysis and data presentation

Sample sizes were based on similar experiments in the literature [23, 44, 53]. All data were analyzed using a two-way ANOVA, performed using IBM SPSS version 24 (SPSS Inc., Chicago, IL). Normality of residuals and variance was assessed using Shapiro–Wilk test and Levene test, respectively. Variance was found to be equal for 16 of 24 datasets. In the eight instances where the assumption of equal variance was violated (eye weight, cornea stroma/endothelial cell count, E18.5 brain Wt-specific mRNA, E18.5 brain Sey-specific mRNA, adult retina Sey-specific mRNA, cornea Sey-specific mRNA, cornea Non-specific mRNA, and blood glucose), we proceeded to run the two-way ANOVA as ANOVA is robust to moderate differences in variability when the sample sizes are equal, as they are in these cases [54]. Main effects of the ANOVA were reported and when significant differences between *Pax6* genotypes and bkgds were found, Tukey’s honest significant difference *post hoc* test was performed. If a significant interaction was discovered, Fisher’s least significant difference *post hoc* test was performed, and Bonferroni’s correction was applied to *α*, to determine where significant interactions occurred. All results are reported as the mean ± standard deviation to reflect the variability within the assays used and the measurements taken. Blood glucose measurements were replicated in two separate cohorts of mice, all other measurements were taken once. Graphs were generated using GraphPad Prism version 6 (GraphPad Software Inc., La Jolla, CA). Each data point is represented by a dot; each genetic background is presented in a different color: B6 (green), F1 (blue), 129 (orange); and each genotype is represented by different shading: Wt (dark), Het (medium), and Hom (light). Error bars represent the standard deviation around the mean for each measure. To facilitate use of this data for benchmarking and power calculations, means and standard deviations for all quantitative measurements were reported in the supplementary tables.

## Results

### Epistasis between the *Pax6* genotype and bkgd influenced eye weight

**Fig. 1:**
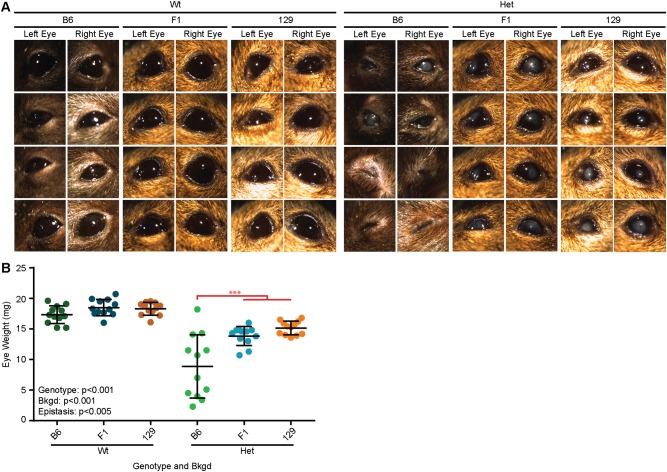
Epistasis produced a severe microphthalmia phenotype in Het B6 eyes. **a** Images of *Pax6*^*+/+*^ (Wt) and *Pax6*^*Sey/+*^ (Het) mouse eyes on three genetic backgrounds (bkgds), C57BL/6J (B6), B6129F1 (F1), and 129S1/SvImJ (129), were examined under a light microscope. Some Het eyes on the B6 bkgd displayed a more severe microphthalmia phenotype where only a very small eye could be detected after manually opening the eyelids with forceps. **b** Weight of enucleated eyes was quantified after fixation. *Pax6* genotype and bkgd were both found to influence eye weight. Epistasis between *Pax6* genotype and bkgd was also discovered, and indicated on the graph (red). *Post hoc* analysis revealed that Het B6 eyes were significantly lighter than Het F1 or 129 eyes (***p < 0.001 for both). Statistical significance was determined using ANOVA, *post hoc* analysis was performed using Fisher’s LSD with Bonferroni’s correction for multiple comparisons. Each dot represents an individual eye and error bars represent the mean ± the standard deviation

The Het phenotype was apparent by visual inspection of adult Wt and Het mice on all three bkgds (Fig. 1a), confirming the stability of the classic phenotype: microphthalmia, central corneal clouding, and corneal vascularization. However, exclusively in B6 mice, a subset of Het mice presented with severe microphthalmia, where the eye was so small that it resembled anophthalmia, as no eye was externally visible, and the eyelids were closed (often a very small eye could be found by manually opening the eyelids). The Het phenotype was quantified by measuring eye weight after enucleation (Table S1). Two-way ANOVA confirmed that *Pax6* genotype and bkgd (*p* < 0.001 for both) both influenced eye weight, where B6 eyes were found to be significantly lighter than F1 and 129 eyes (*p* < 0.001 for both) (Fig. 1b and Fig. S1). Furthermore, a significant epistatic interaction between *Pax6* genotype and bkgd was also found (*p* < 0.005), where B6 Het eyes were significantly smaller than F1 or 129 Het eyes (*p* < 0.001 for both).

Such bkgd-specific differences could have developmental origins, therefore we examined the eyes of Wt, Het, and Hom embryos. As Hom mice only survive until shortly after birth, samples were collected at E18.5. Immediately, the influence of *Pax6* genotype was apparent, as Wt embryos had larger eyes with larger pupils than Het eyes, while Hom embryos presented with anophthalmia and a shortened snout (Fig. S2A). The size of the embryonic whole eye area was estimated using the circumference of the pigmented ring (Table S2). Two-way ANOVA revealed that *Pax6* genotype and bkgd(*p* < 0.001 for both) both significantly influenced embryonic eye size, where 129 embryos had significantly smaller eyes than B6 and F1 embryos (*p* < 0.005 for both) and that B6 embryos had smaller eyes than F1 (*p* < 0.005) (Fig. S2B and Fig. S3). Furthermore, a significant epistatic relationship between *Pax6* genotype and bkgd was also found (*p* < 0.05) where Wt 129 embryos had significantly smaller eyes than Wt B6 (*p* < 0.005) and Wt F1 embryos (*p* < 0.001), and Het F1 embryos had larger eyes than Het 129 embryos (*p* < 0.001). Measurements of Hom eyes were not taken, as no distinguishable ocular structure could be detected. In addition to eye size, a difference in the non-pigmented area, pupil, was also observed by visual inspection. Quantification revealed that *Pax6* genotype and bkgd (*p* < 0.001 for both), where B6 embryos had significantly smaller non-pigmented areas than F1 or 129 embryos (*p* < 0.001 for both) (Fig. S2C). No epistatic interactions were found.

Overall, the gross morphology suggested that a closer inspection of ocular tissues was warranted to determine how these gross differences influence the finer structures of the eye.

### Genetic background influenced retinal thickness

Cryosections from the center of the eyes (defined as cross sections containing the optic nerve) were stained with Hoechst and imaged under a fluorescent microscope. Qualitatively, these images support the gross morphological data, where the reduction in eye weight of Het mice corresponds to a reduction in eye size in all three bkgds (Fig. S4). Additionally, sections of severely microphthalmic eyes revealed major structural perturbations including: anterior synechia, lens hypoplasia or aphakia, and retina dysplasia. The extreme retinal malformations in these eyes made accurate quantification of the retina, and microdissection for RNA and protein extraction, challenging. Thus, such eyes were excluded from the remainder of the study.

**Fig. 2:**
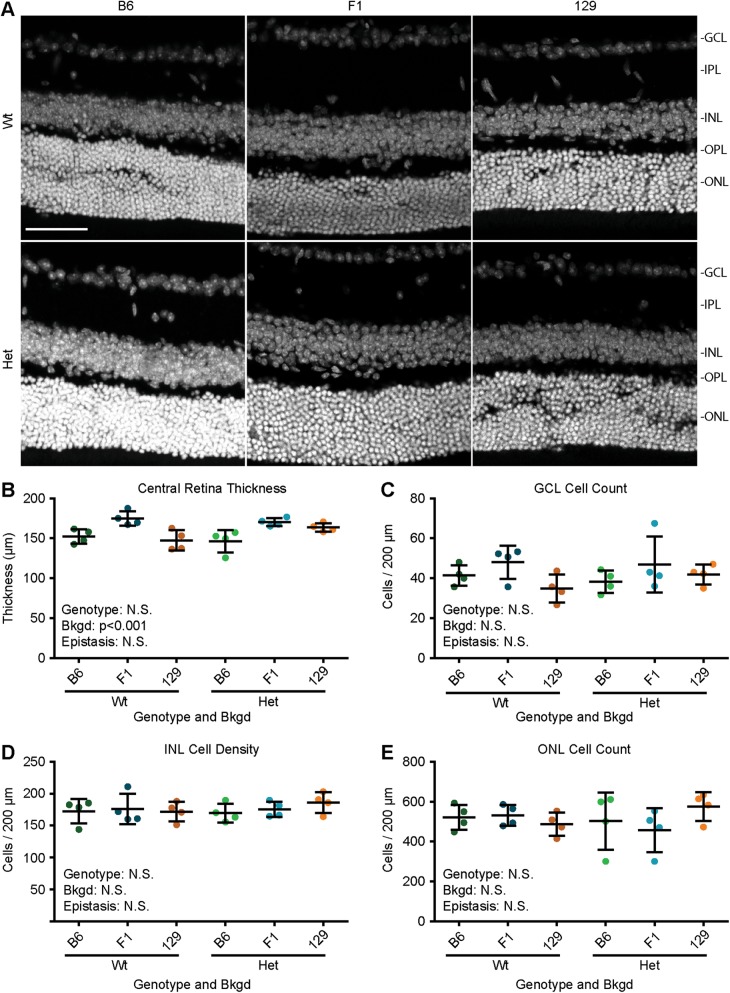
Genetic background, but not *Pax6* genotype, influenced retinal thickness. **a** Confocal scans of *Pax6*^*+/+*^ (Wt) and *Pax6*^*Sey/+*^ (Het) retinas on three genetic backgrounds (bkgds), C57BL/6J (B6), B6129F1 (F1), and 129S1/SvImJ (129). **b** Quantifying retinal thickness revealed that *Pax6* genotype had no influence, but bkgd significantly influenced retinal thickness. Neither *Pax6* genotype nor bkgd were found to influence cell count for the (**c**) ganglion cell layer (GCL), (**d**) inner nuclear layer (INL), or (**e**) outer nuclear layer (ONL). Gray staining (Hoechst), inner plexiform layer (IPL), outer plexiform layer (OPL), not significant (N.S.), scale bar = 50 µm. Statistical significance was determined using ANOVA, *post hoc* analysis was performed using Fisher’s LSD with Bonferroni’s correction for multiple comparisons. Each dot represents an individual eye and error bars represent the mean ± the standard deviation

To explore how *Pax6* genotype and bkgd influence the structure of the retina, confocal images were taken of the central retina (within 500 µm of the optic nerve) (Fig. 2a). Retinal thickness and cell number were quantified (Table S3). ANOVA revealed that bkgd (*p* < 0.005), but not *Pax6* genotype or epistasis, influenced retinal thickness (Fig. 2b), where F1 mice had significantly thicker retinas than B6 (*p* < 0.001) and 129 mice (*p* < 0.005). Cell numbers in each layer were also estimated within a 200 µm window of the central retina, and no significant differences were found in the ganglion cell layer (Fig. 2c), inner nuclear layer (Fig. 2d), or outer nuclear layer (Fig. 2e).

### Epistasis between *Pax6* genotype and genetic background influenced corneal thickness

**Fig. 3:**
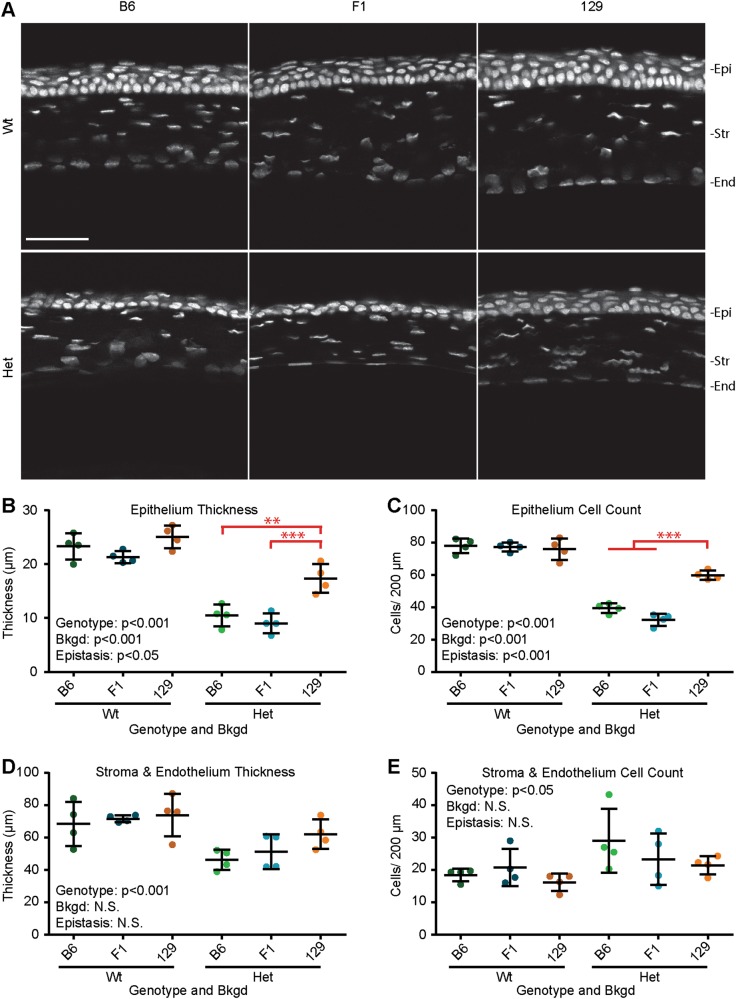
Epistasis influenced corneal epithelial thickness and cell counts. **a** Confocal scans of *Pax6*^*+/+*^ (Wt) and *Pax6*^*Sey/+*^ (Het) corneas on three genetic backgrounds (bkgds), C57BL/6J (B6), B6129F1 (F1), and 129S1/SvImJ (129) were examined. Quantification of (**b**) cornea epithelium (Epi) thickness, (**c**) Epi cell count, (**d**) stroma (Str) plus endothelial (End) thickness, and (**e**) Str plus End cell count, revealed that *Pax6* genotype influenced all measurements. Additionally, epistasis between *Pax6* genotype and bkgd influenced Epi thickness and cell count. *Post hoc* analysis of epistasis, indicated on graphs (red), revealed that Het 129 corneas had significantly thicker Epi than Het B6 and Het F1 corneas (**p < 0.005, and ***p < 0.001, respectively), and that Het 129 corneas had more Epi cells than Het B6 and Het F1 corneas (*p* < 0.001 for both). Gray staining (Hoechst), not significant (N.S.), scale bar = 50 µm. Statistical significance was determined using ANOVA, *post hoc* analysis was performed using Fisher’s LSD with Bonferroni’s correction for multiple comparisons. Each dot represents an individual eye and error bars represent the mean ± the standard deviation

To explore how the structure of the cornea is influenced by *Pax6* genotype and bkgd, confocal images were taken of the central cornea above the pupil, excluding keratolenticular adhesions (Fig. 3a). Measurements of corneal thickness and cell number were taken (Table S4). ANOVA revealed that *Pax6* genotype and bkgd(*p* < 0.001 for both), where 129 mice had thicker cornea epithelia than B6 and F1 mice (*p* < 0.005 and *p* < 0.001, respectively) (Fig. 3b and Fig. S5). Furthermore, a significant epistatic interaction between *Pax6* genotype and bkgd was also found (*p* < 0.05) where Het 129 mice had thicker cornea epithelia than Het B6 (*p* < 0.005) and Het F1 (*p* < 0.001). Similarly, *Pax6* genotype and bkgd influenced the number of cells in the corneal epithelium, where 129 mice had more epithelial cells than B6 and F1 mice (*p* < 0.001 for both) (Fig. 3c and Fig. S6). Furthermore, a significant epistatic interaction between *Pax6* genotype and bkgd was found, where Het 129 mice had more epithelial cells than Het B6 and Het F1 mice (*p* < 0.001 for both).

Examining the corneal stroma and endothelium, ANOVA revealed that *Pax6* genotype (*p* < 0.001), but not bkgd or epistasis, influenced thickness, as the stroma and endothelium of Wt mice was thicker than that of Het mice (Fig. 3d). Conversely, while *Pax6* genotype (*p* < 0.05) also influenced the number of stromal cells, the stroma of eyes from Wt mice had significantly fewer cells than Het mice (Fig. 3e).

### Epistasis between *Pax6* genotype and genetic background influenced *Pax6* mRNA transcript levels

Underlying the ocular phenotype of all Het and Hom mice is PAX6 haploinsufficiency, caused by the *Sey* mutation. Therefore, it could be that differences in PAX6 mRNA and/or protein levels could be the mechanism(s) causing the bkgd-specific differences. This prompted us to investigate the molecular phenotype of these mice. Beginning with mRNA, three RT-ddPCR assays were developed to interrogate *Pax6* mRNA transcript levels. The first two assays specifically amplified either the Wt or *Sey* allele of *Pax6*, while the third was not allele-specific and amplified regions of exons 8 and 9, beyond the *Sey* mutation. As Hom mice do not develop eyes, the brains of E18.5 embryos were used. The same assays were then applied to mRNA from more therapeutically relevant tissues: the adult retina and cornea. Transcript levels for all assays and tissues were reported in Table S5.

**Fig. 4:**
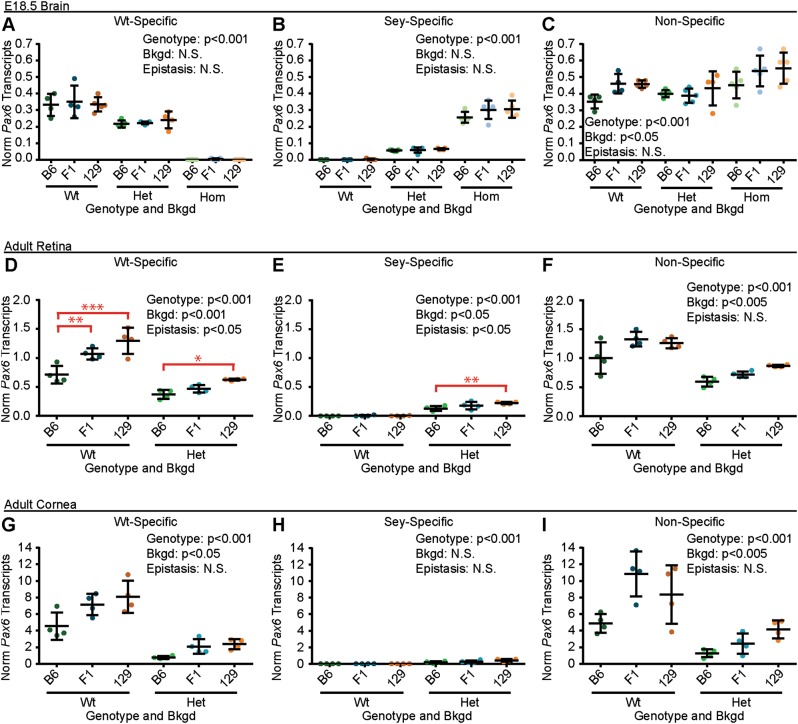
Epistasis influenced adult *Pax6* mRNA levels. *Pax6* mRNA transcripts from (**a**–**c**) embryonic day 18.5 (E18.5) brains, (**d**–**f**) adult retinas, and (**g**–**i**) adult corneas of *Pax6*^*+/+*^ (Wt), *Pax6*^*Sey/+*^ (Het), *Pax6*^*Sey/Sey*^ (Hom; E18.5 brains only), were measured on three genetic backgrounds (bkgds): C57BL/6J (B6), B6129F1 (F1), and 129S1/SvImJ (129). Three RT-ddPCR assays were used to measure Wt (Wt-specific), *Sey* (Sey-specific), and both Wt and *Sey* combined (non-specific) *Pax6* transcript levels. *Pax6*-genotype was found to influence Wt-, Sey-, and Non-specific *Pax6* transcript levels in all tissues. Similarly, bkgd influenced at least one *Pax6* transcript levels in all tissues. Epistasis between *Pax6* genotype and bkgd was also measured in retinal mRNA samples, and indicated on the graphs (red). For Wt-specific *Pax6* transcripts, *post hoc* analysis revealed that retinas from Wt B6 mice had lower transcript levels than retinas from Wt F1 and Wt 129 mice (**p < 0.005 and ***p < 0.001; respectively) and retinas from Het B6 mice had significantly lower Wt-specific *Pax6* transcript levels than retinas from Het 129 mice (*p* < 0.05). *Post hoc* analysis of Sey-specific *Pax6* transcripts also revealed that retinas from Het B6 mice had lower Sey-specific *Pax6* transcript levels than retinas from 129 mice (*p* < 0.005). Not significant (N.S.). Statistical significance was determined using ANOVA, *post hoc* analysis was performed using Fisher’s LSD with Bonferroni’s correction for multiple comparisons. Each dot represents an individual mouse and error bars represent the mean ± the standard deviation

In the E18.5 brain, ANOVA revealed that *Pax6* genotype (*p* < 0.001), but not bkgd or epistasis, influenced Wt-specific *Pax6* transcript levels (Fig. 4a), where Wt embryos had significantly higher Wt-specific *Pax6* transcript levels than brains from Het embryos (*p* < 0.001) and brains from both Wt and Het embryos had significantly higher levels than brains from Hom embryos (*p* < 0.001 for both). *Pax6* genotype alone (*p* < 0.001), also significantly influenced the amount of *Sey*-specific mRNA levels (Fig. 4b), where Wt embryos had significantly fewer *Sey* transcripts than Het embryos (*p* < 0.005) and that both Wt and Het embryos had significantly fewer *Sey* transcripts than Hom embryos (*p* < 0.001). Similarly, *Pax6* genotype (*p* < 0.001) and bkgd (*p* < 0.05), both significantly influenced non-specific *Pax6* mRNA levels (Fig. 4c), where Hom embryos had significantly higher *Pax6* transcript levels than Wt and Het embryos (*p* < 0.001 for both). Additionally, it was found that B6 embryos had significantly fewer *Pax6* transcripts than brains from 129 embryos (*p* < 0.05).

In the adult retina, *Pax6* genotype and bkgd (*p* < 0.001 for both) both significantly influenced Wt-specific *Pax6* transcript levels (Fig. 4d and Fig. S7), where B6 mice had significantly lower Wt-specific *Pax6* mRNA levels than 129 retinas (*p* < 0.01). Furthermore, a significant epistatic interaction between *Pax6* genotype and bkgd was found (*p* < 0.05), where Wt B6 mice had significantly lower Wt-specific *Pax6* transcript levels than retinas from Wt F1 and Wt 129 mice (*p* < 0.005 and *p* < 0.001, respectively), and that retinas from Het B6 mice had significantly lower Wt-specific *Pax6* transcript levels than retinas from Het 129 mice (*p* < 0.05). Similarly, *Pax6* genotype (*p* < 0.001) and bkgd (*p* < 0.05) both significantly influenced *Sey*-specific *Pax6* transcript levels, revealing B6 mice had lower *Sey*-specific *Pax6* transcript levels than retinas from 129 mice (*p* < 0.05) (Fig. 4e and Fig. S8). Furthermore, a significant epistatic interaction between *Pax6* genotype and bkgd was also found (*p* < 0.05), revealing that retinas from Het B6 mice had lower *Sey-*specific *Pax6* transcript levels than retinas from Het 129 mice (*p* < 0.005). Finally, *Pax6* genotype (*p* < 0.001) and bkgd (*p* < 0.005), but not epistasis, influenced non-specific *Pax6* transcript levels (Fig. 4f), revealing that retinas from Het B6 mice had lower non-specific *Pax6* transcript levels than retinas from Het F1 (*p* < 0.05) and Het 129 (*p* < 0.005).

In the adult cornea, similar patterns to the retina were observed, where *Pax6* genotype (*p* < 0.001), and bkgd (*p* < 0.05), but not epistasis, influenced Wt-specific *Pax6* transcript levels, from F1 (*p* < 0.005) and 129 mice (*p* < 0.05) (Fig. 4g). *Pax6* genotype (*p* < 0.001), but not bkgd or epistasis, influenced the levels of *Sey-*specific *Pax6* transcripts (Fig. 4h). *Pax6* genotype (*p* < 0.001) and bkgd (*p* < 0.005), but not epistasis, influenced non-specific *Pax6* transcript levels, revealing that B6 mice had lower non-specific *Pax6* transcript levels than corneas from 129 mice (*p* < 0.005) (Fig. 4i).

### *Pax6* genotype influenced PAX6 protein levels

**Fig. 5:**
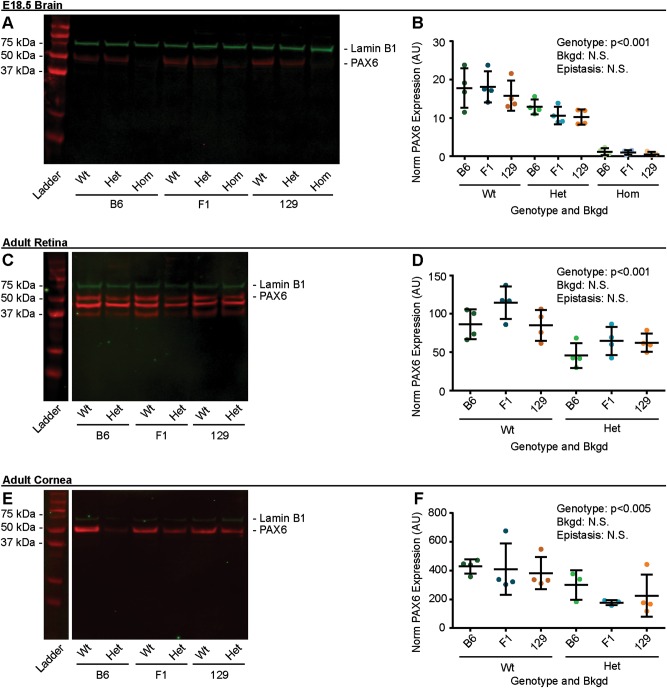
Only *Pax6* genotype influenced PAX6 protein levels. PAX6 protein from **a** and **b** embryonic day 18.5 (E18.5) brains, **c** and **d** adult retinas, and **e** and **f** adult corneas of *Pax6*^*+/+*^ (Wt), *Pax6*^*Sey/+*^ (Het), *Pax6*^*Sey/Sey*^ (Hom; E18.5 brains only), was measured by western blot on three genetic backgrounds (bkgds): C57BL/6J (B6), B6129F1 (F1), and 129S1/SvImJ (129). Genotype was found to influence PAX6 protein levels in all tissues examined. PAX6 immunostaining (red), Lamin B1 immunostaining (green), arbitrary units (AU), not significant (N.S.). Statistical significance was determined using ANOVA. Each dot represents an individual mouse and error bars represent the mean ± the standard deviation

As bkgd influenced *Pax6* mRNA transcript levels, we proceeded to determine if those differences translated to the protein level using western blotting. As expected, blots of protein samples from E18.5 mouse brains reveled a qualitative reduction in PAX6 band intensity between Wt, Het, and Hom mice (Fig. 5a). Western blots were quantified (Table S6), and ANOVA revealed *Pax6* genotype (*p* < 0.001), but not bkgd or epistasis, influenced PAX6 protein levels (Fig. 5b), revealing that brains from Wt embryos had more protein than brains from Het and Hom embryos (*p* < 0.001 for both). Similarly, protein samples taken from adult mouse retinas (Fig. 5c, d), and corneas (Fig. 5e, f) revealed that *Pax6* genotype (*p* < 0.001 and *p* < 0.005, respectively), but not bkgd or epistasis influenced PAX6 protein levels, and that samples from Wt retina and corneas had more PAX6 protein than retinas and corneas from Het mice.

### Epistasis between *Pax6* genotype and bkgd influenced blood glucose levels

**Fig. 6:**
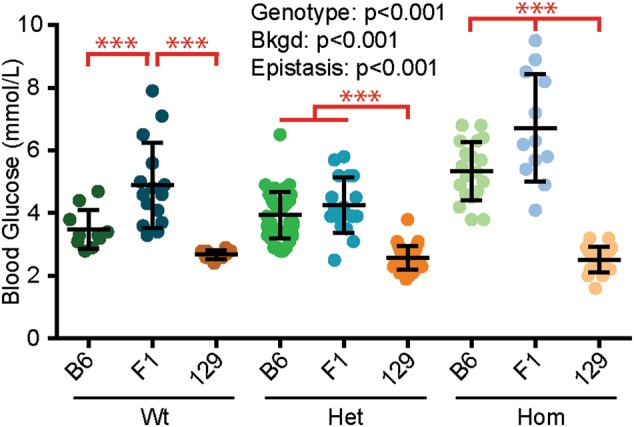
Epistasis influenced embryonic blood glucose levels. Blood glucose from *Pax6*^*+/+*^ (Wt), *Pax6*^*Sey/+*^ (Het), *Pax6*^*Sey/Sey*^ (Hom) embryonic day 18.5 brains was measured on three genetic backgrounds (bkgds): C57BL/6J (B6), B6129F1 (F1), and 129S1/SvImJ (129). Genotype and bkgd was found to influence blood glucose levels. Epistasis between *Pax6* genotype and bkgd also influenced blood glucose levels, as indicated on graph (red). *Post hoc* analysis revealed that Wt F1 embryos had significantly higher blood glucose levels than Wt B6 and Wt 129 embryos (***p < 0.001 for both). It was also revealed that Het 129 embryos had significantly lower blood glucose levels than Het B6 and Het F1 embryos (*p* < 0.001 for both). Finally, Hom F1 embryos had significantly higher blood glucose levels than Hom B6 and Hom 129 embryos (*p* < 0.001), and Hom B6 embryos had higher blood glucose levels than Wt 129 embryos (*p* < 0.001). Statistical significance was determined using ANOVA, *post hoc* analysis was performed using Fisher’s LSD with Bonferroni’s correction for multiple comparisons. Each dot represents an individual embryo and error bars represent the mean ± the standard deviation

To quantify how the *Sey* mutation influences pancreas function, blood glucose measurements were taken from E18.5 Wt, Het, and Hom mouse embryos from each bkgd (Table S7). *Pax6* genotype and bkgd, were both found to significantly influence blood glucose levels, revealing that Hom embryos had higher blood glucose levels than Wt (*p* < 0.005) and Het embryos (*p* < 0.001), F1 embryos had higher blood glucose levels than B6 and 129 (*p* < 0.001 for both), and that B6 embryos had higher blood glucose levels than 129 embryos (*p* < 0.001) (Fig. 6 and Fig. S8). Furthermore, a significant epistatic interaction between *Pax6* genotype and bkgd was found (*p* < 0.001), revealing that Wt F1 embryos had higher blood glucose levels than Wt B6 and Wt 129 embryos (*p* < 0.001 for both), Het 129 embryos had significantly lower blood glucose levels than Het B6 and Het F1 embryos (*p* < 0.001 for both), Hom F1 embryos had higher blood glucose levels than Hom B6 and Hom 129 embryos (*p* < 0.001 for both), and Hom B6 embryos had higher blood glucose levels than Wt 129 embryos (*p* < 0.001).

## Discussion

We have contributed substantially to the general knowledge of *PAX6* null alleles by performing a systematic examination of the Het and Hom phenotypes across multiple bkgds. Consistently, the *Sey* allele produced microphthalmia, decreased corneal epithelial thickness, reduced ocular *Pax6* mRNA levels, and reduced PAX6 protein levels. Surprisingly, we made the novel discovery that the *Sey* allele does not decrease retinal thickness in Het mice, conflicting with previous descriptions of Het B6 eyes [23, 24]. This could be due to our exclusion of Het B6 eyes with severe microphthalmia, where extreme retinal malformations are apparent upon histological examination, making accurate quantification challenging. Our study also revealed thinning of the corneal stroma in Het eyes. Conversely, a previous study reported stromal thickening in Het mice carrying the *Pax6*^*Sey-Neu*^ allele on an unspecified background. However, the authors of that study questioned if this was a biological finding, or a technical artifact [18].

Typically, mRNA transcripts containing premature termination codons are degraded by nonsense mediated decay (NMD) [55], and *Pax6* is known to be subject to NMD in both humans [56–58] and mice [59]. It is known that compensatory mechanism can help rescue deleterious mutations [60–62]. Specifically, previous in situ hybridization studies detected *Pax6* mRNA in Hom *Sey* brains [63, 64]. Our results support NMD for *Pax6* in the adult retina and cornea. Surprisingly, we discovered this was not the case in embryonic brains, where Hom embryos had the highest levels of *Pax6* mRNA. One explanation for the elevated levels of *Sey* transcripts in the embryonic mouse brains might be suppression of NMD [65, 66]. However, the Hom embryos had approximately five times more *Sey* transcripts than Het embryos, which suggests that there is a mechanism beyond NMD suppression. Interestingly, in Het embryos, Wt-specific *Pax6* mRNA levels were also higher than expected; greater than 50% of Wt embryo levels. Together, these findings suggest that a compensatory mechanism is acting upon Wt and *Sey* alleles or transcripts, boosting *Pax6* transcription or transcript levels during the development of Het and Hom embryos. While PAX6 autoregulation has been previously reported [67], this does not seem to be the mechanism observed here, since Hom mice with no functional PAX6, have the highest transcript levels [65, 66]. Therefore, we hypothesize that a development-specific mechanism, which does not depend on functional PAX6, drives compensatory *Pax6* transcription in embryonic mouse brains.

In all tissues tested, the influence of mouse bkgd was detected, resulting in differences in eye weight, retinal thickness, corneal thickness, *Pax6* mRNA levels, and blood glucose levels. In the eye, the B6 bkgd introduced considerable variability, with eyes varying from severe to moderate microphthalmia and severe to moderate structural perturbations. As microphthalmia is very rarely reported in aniridia [68–70], and in some cases is thought to be caused by mutations in other important eye genes such as *SOX2* and *OTX2* in addition to the aniridia causing *PAX6* mutation [71, 72]. Therefore, severe microphthalmia is not an ideal phenotype to study for treatment of aniridia. Furthermore, such a severe phenotype, that does not mimic typical aniridia, represents a danger to therapeutic development. The increased variability can bias results, drive up experimental cohorts, and threaten reproducibility, underscoring why the choice of bkgd is critical.

Our discovery of epistasis between *Pax6* genotype and the B6 and 129 bkgds, producing severe microphthalmy and suppressed blood glucose levels, respectively, is congruent with earlier reports that Wt C57BL/6 mice spontaneously develop microphthalmia [73], and that Wt 129 Sv mice have low blood glucose levels [74]. Interestingly, crossing B6 dams and 129 sires normalized these severe phenotypes, suggesting that the cause lay in the recessive homozygous alleles in the B6 and 129 bkgds, and that these phenotypes, a consequence of the unnatural inbred strains, are not likely to represent human aniridia. To this point, of the seven epistatic interactions measured where one bkgd differed significantly from two non-significantly different bkgds, only one involved F1 mice as the variant. Therefore, in the development of new therapeutics for aniridia, where a phenotype that is caused by the *Pax6* mutation is desired, unobstructed by interaction with bkgd, we recommend using the genetically defined B6129F1 hybrid bkgd. As epistasis with inbred strains may confound the development of other therapeutics for other disorders too, the use of the defined hybrid B6129F1 mice, or indeed other hybrids, may broadly benefit the field.

Our results provide a description of epistasis between *Pax6* and three commonly used bkgds. The discovery of epistasis between *Pax6* genotype and B6 bkgd, presents a research opportunity to explore the factors that incline ocular development towards a moderate or severe phenotype in genetically identical eyes. Previous modifier studies for other genes have revealed that members of the same gene family can act as modifiers [75]. As other *PAX* transcription factors are involved in ocular development [76], we hypothesize that other *PAX* genes may be good initial candidates to modify the *Sey* phenotype.

Aniridia is often diagnosed after birth, when iris hypoplasia is easily observed [14, 77]. Therefore, important therapeutic timepoints for aniridia are the juvenile and adult eye. However, many descriptions of the *Sey* phenotype focus on the developing *Sey* eye and phenotypic descriptions of the adult *Pax6* haploinsufficiency mouse come from diverse sources that are spread across numerous *Pax6* mutations and bkgds [18, 22–24, 78]. In this context, our systematic evaluation of the Het phenotype, across three bkgds, bolsters the data available upon which evidence-based decisions can be made when evaluating new therapeutics for aniridia. We further think the B6129F1-*Sey* model may be useful in defining new human-relevant outcome measures for future therapeutics.

## Electronic supplementary material

Supplementary Materials
