# Correction: Epistasis between Pax6^Sey^ and genetic background reinforces the value of defined hybrid mouse models for therapeutic trials

**DOI:** 10.1038/s41434-018-0054-3

**Published:** 2019-01-02

**Authors:** Jack W. Hickmott, Uvini Gunawardane, Kimberly Jensen, Andrea J. Korecki, Elizabeth M. Simpson

**Affiliations:** 1 Centre for Molecular Medicine and Therapeutics at BC Children’s Hospital, University of British Columbia, Vancouver, BC Canada; 2 Department of Medical Genetics, University of British Columbia, Vancouver, BC Canada; 3 Department of Psychiatry, University of British Columbia, Vancouver, BC Canada; 4 Department of Ophthalmology and Visual Science, University of British Columbia, Vancouver, BC Canada

**Keywords:** Genetics, Visual system

Correction to: Gene Therapy;

10.1038/s41434-018-0043-6; published online 26 Sept 2018

This Article was originally published under Nature Research’s License to Publish, but has now been made available under a CC BY 4.0 license. The PDF and HTML versions of the Article have been modified accordingly.

