# Supplementary material for: Epistasis between Pax6Sey and genetic background reinforces the value of defined hybrid mouse models for therapeutic trials
Source: Gene Ther. 2018 Sep 26;25(8):524–37. doi: 10.1038/s41434-018-0043-6 (PMC6335240; doi:10.1038/s41434-018-0043-6)
Supplement: Supplementary file 1 — Supplementary Materials [file 41434_2018_43_MOESM1_ESM.pdf]

Supplementary Materials

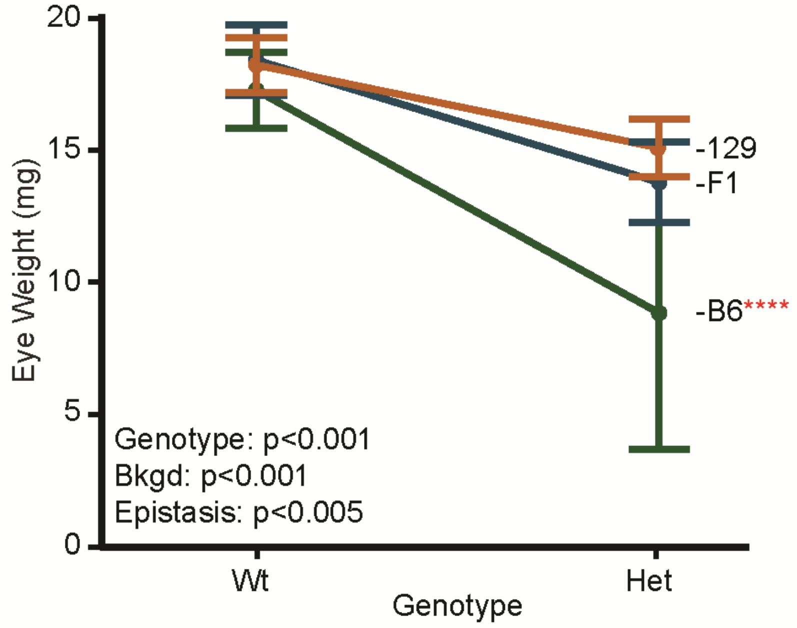

Figure S1. Genotype, genetic background (bkgd), and epistatic interactions influence mouse eye weight (raw data presented in Figure 1B).

The influence of *Pax6* genotype is present as the weight of *Pax6*$^{+/+}$ (Wt) eyes is larger than the weight of *Pax6*$^{Sey/+}$ (Het) eyes ($p<0.001$). The influence of bkgd is present, as the green line (denoting B6 mice) is significantly lower than the blue and orange lines (F1 and 129 respectively) ($p<0.001$). The influence of epistasis is present, as the slope of the green line is significantly (****, $p<0.001$) non-parallel with the slopes of the blue and orange lines. This indicates that the B6 bkgd interacts with the *Sey* allele, exacerbating the eye weight phenotype, resulting in even lighter eyes in Het B6 mice than would be expected from the Het genotype or B6 background separately. Error bars represent standard deviation.

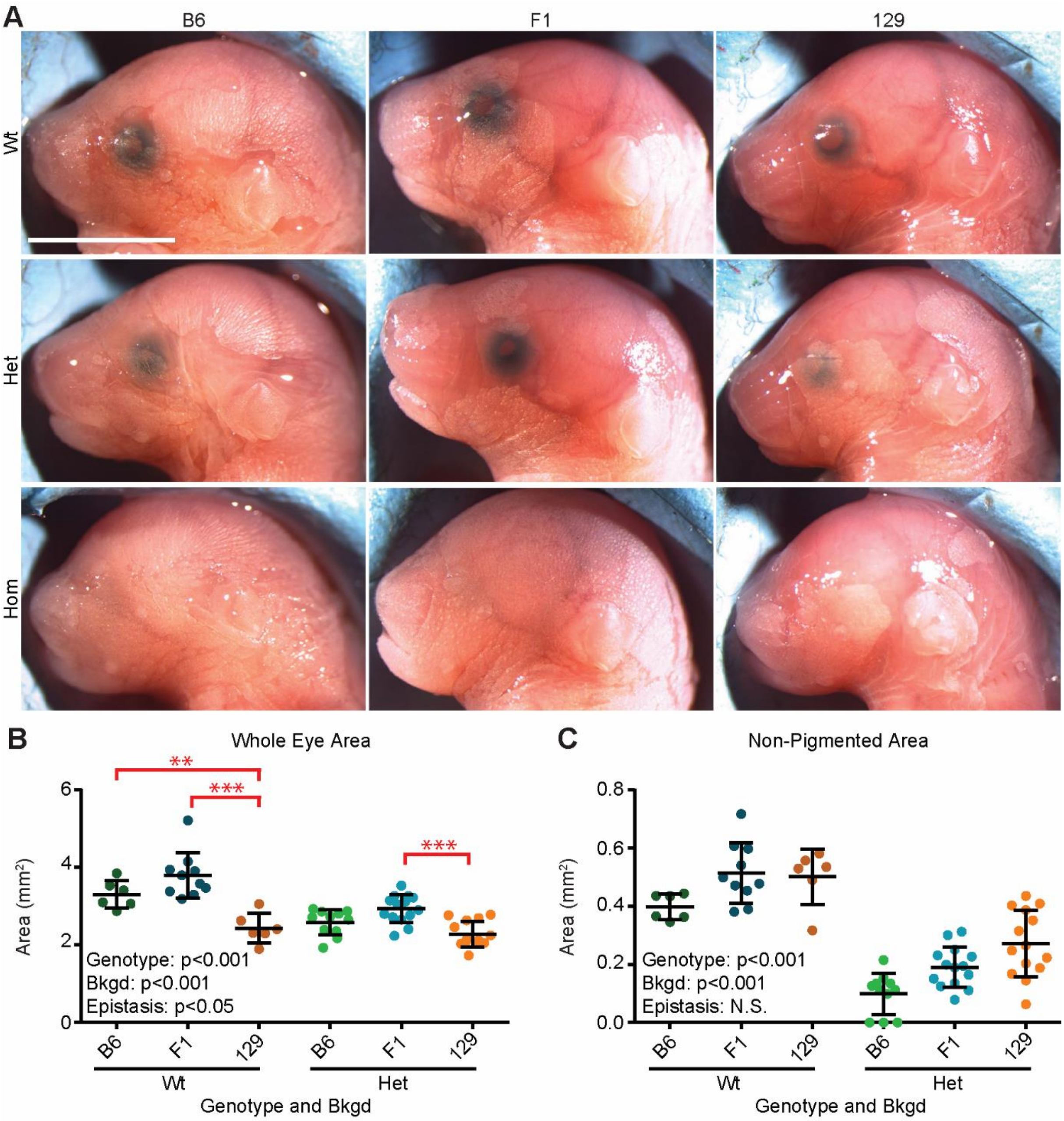

Figure S2. Epistasis influenced the size of embryonic mouse whole eye area. A) Images of *Pax6*$^{+/+}$ (Wt), *Pax6*$^{Sey/+}$ (Het), and *Pax6*$^{Sey/Sey}$ (Hom) embryonic day 18.5 mouse embryos on three genetic backgrounds (bkgd): C57BL/6J (B6), B6129F1 (F1), and 129S1/SvImJ (129), revealed microphthalmia and anophthalmia in Het and Hom embryos respectively. Quantification of **B)** whole eye area and **C)** non-pigmented area, reveals that both *Pax6* genotype and bkgd influenced eye size. Furthermore, epistasis between *Pax6* genotype and bkgd was detected, and indicated on the graph (red). *Post hoc* analysis

revealed that Wt 129 embryos had significantly smaller eyes than Wt B6 (**, $p<0.005$) and F1 (***, $p<0.001$), and that Het F1 embryos had significantly larger eyes than Het 129 embryos ($p<0.001$). Statistical significance was determined using ANOVA, *post hoc* analysis was performed using Fisher's LSD with Bonferroni's correction for multiple comparisons. Each circle represents an individual embryo and error bars represent the mean ± the standard deviation.

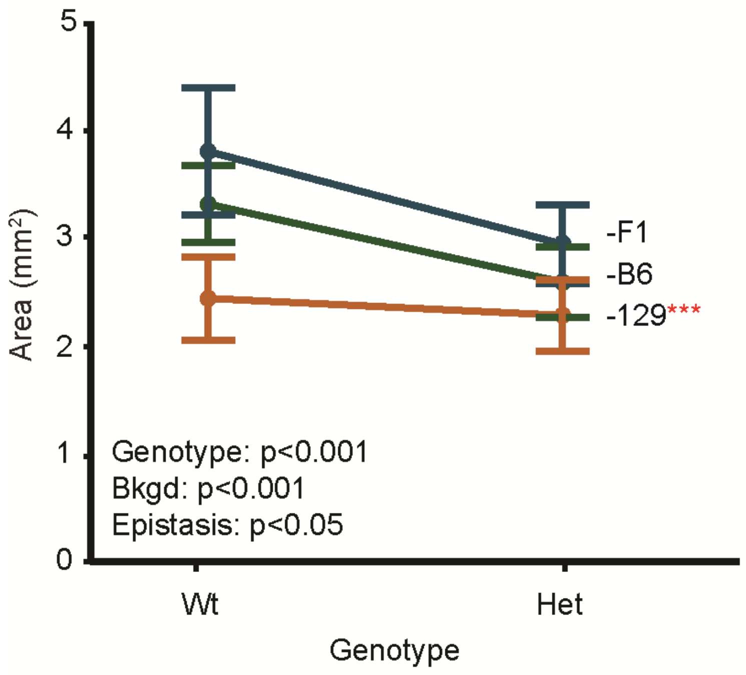

Figure S3. Genotype, genetic background (bkgd), and epistatic interactions influenced embryonic mouse eye area (raw data presented in Figure S2B). The influence of *Pax6* genotype is present as the area of *Pax6*$^{+/+}$ (Wt) eyes is larger than the area of *Pax6*$^{Sey/+}$ (Het) eyes ($p<0.001$). The influence of bkgd is present, as the orange line (denoting 129 mice) is significantly lower than the green and blue lines (B6 and F1 respectively) ($p<0.001$). The influence of epistasis is present, as the slope of the orange line is significantly (***, $p<0.005$) non-parallel with the slopes of the green and blue lines. This indicates that the 129 bkgd interacts with the *Pax6* allele, resulting in smaller embryonic eyes in Wt 129 mice than would be expected from the Wt genotype or 129 bkgd. Error bars represent standard deviation.

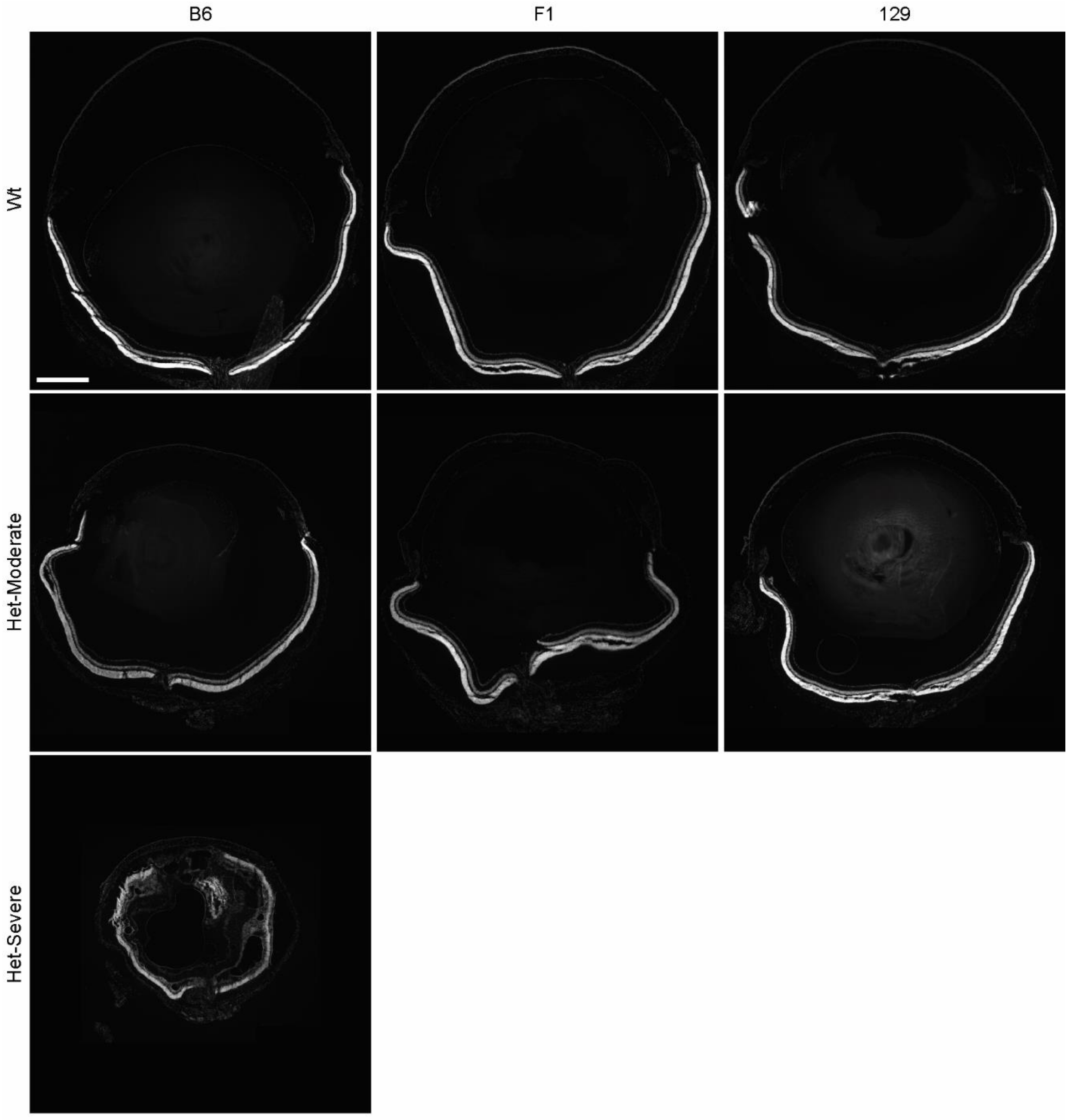

Figure S4. Retinal, lens, and corneal structural abnormalities evident in severely microphthalmic eyes. Tiled images of Hoechst stained cryosections from *Pax6*$^{+/+}$ (Wt) and *Pax6*$^{Sey/+}$ (Het) C57BL/6J (B6), B6129F1 (F1), and 129S1/SvImJ (129) eyes revealed that despite a reduction in size, the general structure of the Het eye is intact (Het-Moderate). However, in a subset of Het B6 eyes with severe microphthalmia, major structural abnormalities in the retina, lens, and cornea were apparent (Het-Sever). Scale bar = 500 µm.

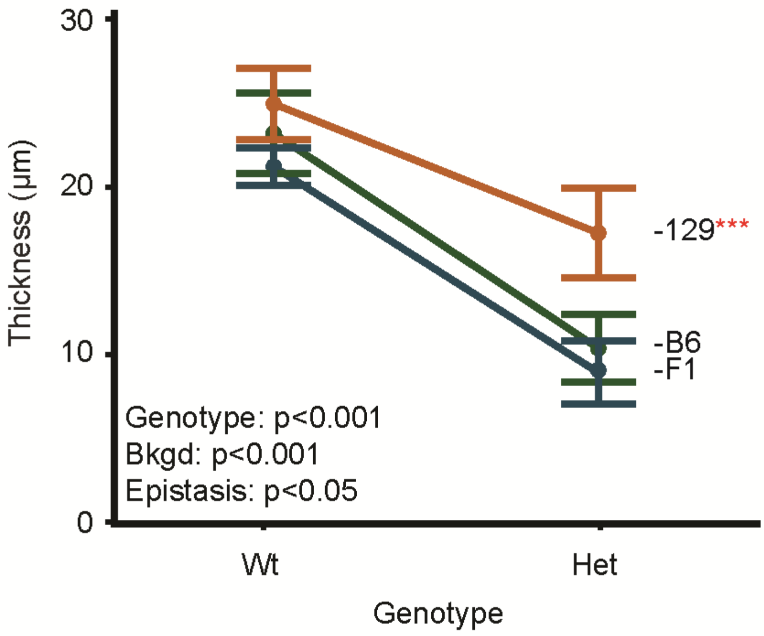

Figure S5. Pax6 genotype, genetic background (bkgd), and epistatic interactions influenced cornea epithelial thickness (raw data presented in Figure 3B). The influence of *Pax6* genotype is present, as the mean corneal epithelial thickness of *Pax6*$^{+/+}$ (Wt) eyes is greater than the corneal epithelial thickness of *Pax6*$^{Sey/+}$ (Het) eyes. The influence of bkgd is present, as the orange line (denoting 129 mice) is higher than the green and blue lines (B6 and F1 mice respectively). The influence of epistasis is present, as the slope of the orange line is significantly (***, $p<0.005$) non-parallel with the slopes of the green and blue lines. This indicates that the 129 bkgd interacts with *the* Sey allele, moderating the corneal epithelial thickness phenotype, resulting in a thicker cornea epithelium in Het 129 mice than would be expected from the Het genotype or 129 bkgd separately. Error bars represent standard deviation.

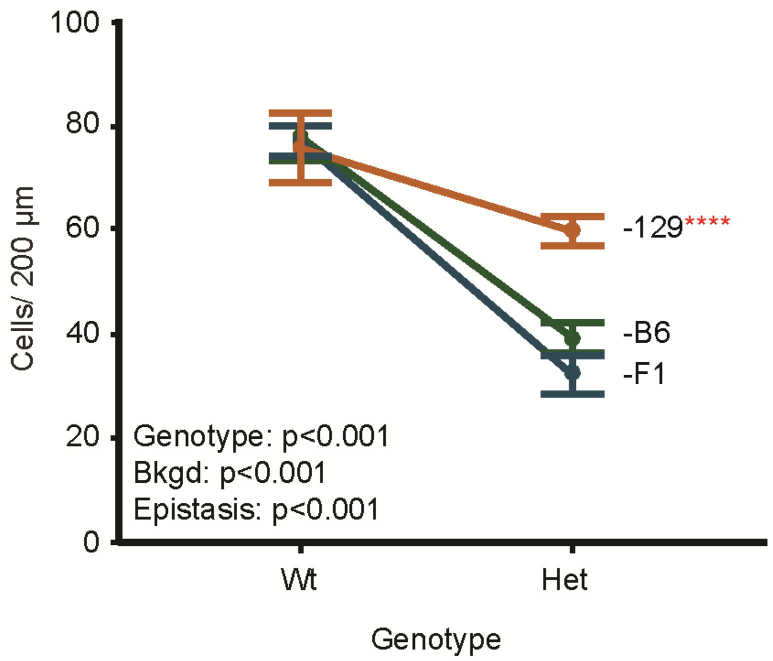

Figure S6. Pax6 genotype, genetic background (bkgd), and epistatic interactions influenced cornea epithelial cell numbers (raw data presented in Figure 3C). The influence of *Pax6* genotype is present, as the mean corneal epithelial cell count of *Pax6*$^{+/+}$ (Wt) eyes is greater than the corneal epithelial thickness of *Pax6*$^{Sey/+}$ (Het) eyes. The influence of bkgd is present, as the orange line (denoting 129 mice) is higher than the green and blue lines (B6 and F1 mice respectively). The influence of epistasis is present, as the slope of the orange line is significantly (****, $p<0.001$) non-parallel with the slopes of the green and blue lines. This indicates that the 129 bkgd interacts with *the* Sey allele, moderating the corneal epithelial cell count phenotype, resulting in more corneal epithelial cells in Het 129 Het mice than would be expected from the Het genotype or 129 bkgd separately. Error bars represent standard deviation.

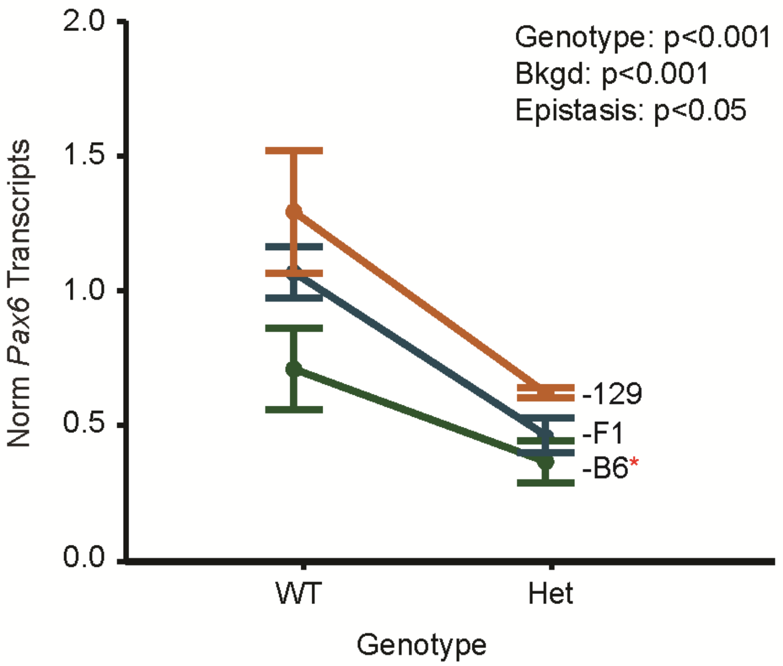

Figure S7. Pax6 genotype, genetic background (bkgd), and epistatic interactions influenced retinal *Pax6* Wt-specific mRNA levels (raw data presented in Figure 4D). The influence of *Pax6* genotype is present, as the mean Wt-specific mRNA levels of *Pax6*$^{+/+}$ (Wt) eyes is greater than the mean Wt-specific mRNA levels of *Pax6*$^{Sey/+}$ (Het) eyes. The influence of bkgd is present, as the orange line (denoting 129 mice) is higher than the green and blue lines (B6 and F1 mice respectively). The influence of epistasis is present, as the slope of the green line is significantly (*, p<0.05) non-parallel with the slopes of the blue and orange lines. This indicates that the B6 bkgd interacts with the *Pax6* allele, reducing the amount of Wt-specific mRNA in Wt B6 mice than would be expected from the Wt genotype or B6 bkgd separately. Error bars represent standard deviation.

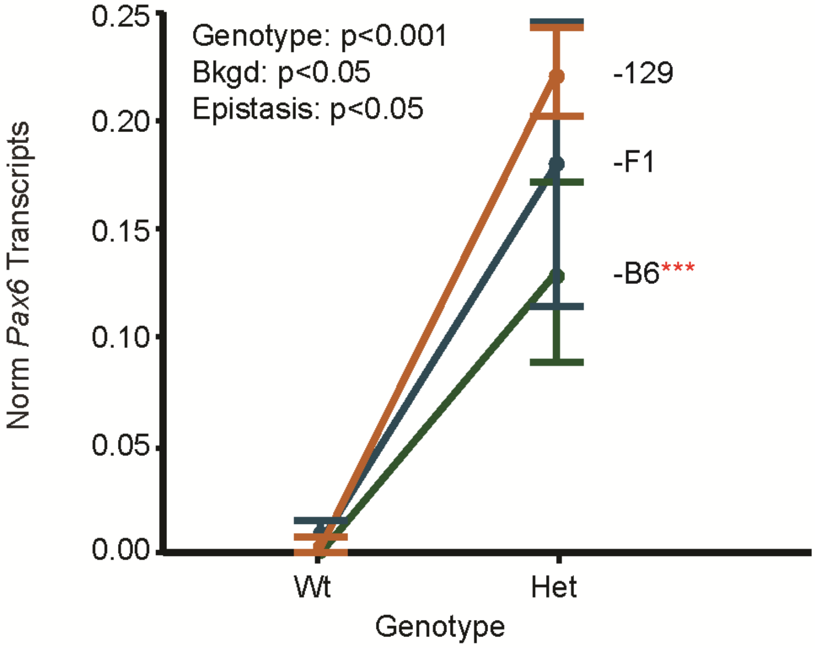

Figure S8. Pax6 genotype, genetic background (bkgd), and epistatic interactions influenced retinal *Pax6 Sey*-specific mRNA levels (raw data presented in Figure 4E). The influence of *Pax6* genotype is present, as the mean *Sey*-specific mRNA levels of *Pax6*$^{+/+}$ (Wt) eyes is lower than the mean Sey-specific mRNA levels of *Pax6*$^{Sey/+}$ (Het) eyes. The influence of bkgd is present, as the green line (denoting B6 mice) is significantly lower than the orange line (129 mice). The influence of epistasis is present, as the slope of the green line is significantly (***, $p<0.005$) non-parallel with the slope of the orange line. This indicates that the B6 bkgd interacts with the *Sey* allele, reducing the amount of Sey-specific mRNA in Het B6 mice than would be expected from the Het genotype or B6 bkgd separately. Error bars represent standard deviation.

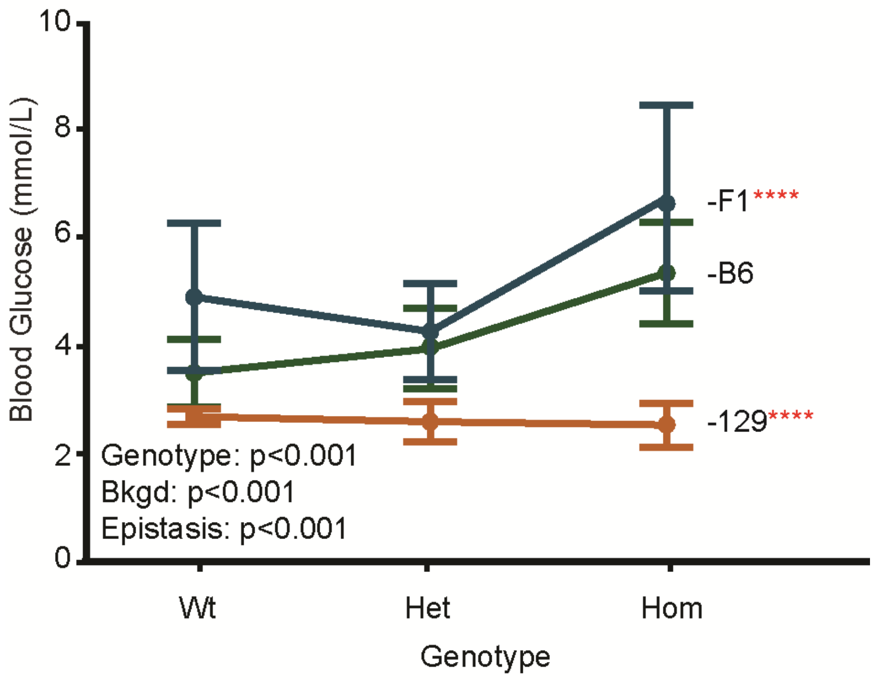

Figure S9. Pax6 genotype, genetic background (bkgd), and epistatic interactions influenced embryonic blood glucose levels (raw data presented in Figure 6). The influence of *Pax6* genotype is present, as the mean blood glucose levels of *Pax6*$^{+/+}$ (Wt) and *Pax6*$^{Sey/+}$ (Het) embryos is lower than the mean blood glucose levels of *Pax6*$^{Sey/Sey}$ (Hom) embryos. The influence of bkgd is present, as the blue line (denoting F1 embryos) is significantly higher than the green and orange lines (B6 and 129 embryos, respectively) and the green line is significantly higher than the orange line The influence of epistasis is present, as the slope of the orange line is significantly (****, p<0.001) non-parallel with the slopes of the green and blue lines. This indicates that the 129 bkgd interacts with the *Pax6* and *Sey* alleles, reducing the blood glucose levels of Het and Hom embryos in a way not expected from just the from the Het and Hom genotypes, or 129 bkgd separately. Additionally, the slope of the blue line is significantly (p<0.001) non-parallel with that of the green line. This indicates that the F1 bkgd interacts with the *Pax6* and *Sey* alleles, increasing the blood glucose levels in a way not expected from the just the Wt or Het genotypes, or F1 background separately. Error bars represent standard deviation.

Table S1. Mean and relative adult eye weights

**Bkgd**	**Eye Weight (mg)**		
	Wt	Het	Het/Wt
B6	17.3 ± 1.43	8.88 ± 5.18	0.51
F1	18.5 ± 1.33	13.8 ± 1.54	0.75
129	18.3 ± 1.04	15.2 ± 1.11	0.83

Pax6$^{+/+}$ (Wt), *Pax6*$^{Sey/+}$ (Het), ratio of Het measurement to Wt measurement (Het/Wt), genetic background (bkgd), C57BL/6J (B6), B6129F1 (F1), 12921/SvImJ (129). Data is reported as mean ± standard deviation.

Table S2. Mean and relative embryonic eye size

Bkgd	Whole Eye Area (mm^2)		
	Wt	Het	Het/Wt
B6	3.31 ± 0.15	2.58 ± 0.10	0.78
F1	3.79 ± 0.19	2.94 ± 0.10	0.78
129	2.43 ± 0.16	2.28 ± 0.09	0.94
**Bkgd**	**Non-Pigmented Area (mm^2)**		
	Wt	Het	Het/Wt
B6	0.40 ± 0.04	0.10 ± 0.07	0.25
F1	0.51 ± 0.10	0.19 ± 0.07	0.38
129	0.51 ± 0.10	0.27 ± 0.11	0.53

Pax6$^{+/+}$ (Wt), *Pax6*$^{Sey/+}$ (Het), ratio of Het measurement to Wt measurement (Het/Wt), genetic background (bkgd), C57BL/6J (B6), B6129F1 (F1), 12921/SvImJ (129). Data is reported as mean ± standard deviation.

Table S3. Mean and relative adult retina thickness and cell counts

Bkgd	RetinalThickness (µm)		
	Wt	Het	Het/Wt
B6	152.41 ± 8.93	146.30 ± 14.19	0.96
F1	174.97 ± 9.04	170.30 ± 5.08	0.97
129	147.40 ± 12.7	163.70 ± 5.36	1.11
**Bkgd**	**GCL Cell Count (cells/200 µm of retina)**		
	Wt	Het	Het/Wt
B6	41.4 ± 5.12	38.3 ± 5.57	0.93
F1	48.0 ± 8.30	47.0 ± 14.0	0.98
129	34.8 ± 7.02	41.8 ± 5.00	1.18
**Bkgd**	**INL Cell Count (cells/200 µm of retina)**		
	Wt	Het	Het/Wt
B6	173 ± 19.3	170 ± 14.6	0.98
F1	176 ± 24.0	176 ± 12.1	1.00
129	172 ± 15.5	186 ± 16.3	1.08
**Bkgd**	**ONL Cell Count (cells/200 µm of retina)**		
	Wt	Het	Het/Wt
B6	521 ± 63.0	503 ± 143	0.97
F1	531 ± 52.2	458 ± 110	0.86
129	487 ± 58.5	576 ± 72.1	1.18

Pax6$^{+/+}$ (Wt), *Pax6*$^{Sey/+}$ (Het), ratio of Het measurement to Wt measurement (Het/Wt), genetic background (bkgd), C57BL/6J (B6), B6129F1 (F1), 12921/SvImJ (129). Data is reported as mean ± standard deviation.

Table S4. Mean and relative adult cornea thickness and cell counts

Bkgd	EpiThickness (µm)		
	Wt	Het	Het/Wt
B6	23.3 ± 2.42	10.5 ± 2.01	0.45
F1	21.3 ± 1.10	9.02 ± 1.86	0.42
129	25.1 ± 2.12	17.4 ± 2.69	0.69
**Bkgd**	**Epi Cell Count (cells/200 µm of cornea)**		
	Wt	Het	Het/Wt
B6	78.1 ± 4.56	39.4 ± 2.99	0.45
F1	77.3 ± 2.84	32.3 ± 3.72	0.42
129	75.9 ± 6.66	59.9 ± 2.79	0.79
**Bkgd**	**Str and End Thickness (µm)**		
	Wt	Het	Het/Wt
B6	68.6 ± 13.7	46.3 ± 6.20	0.68
F1	71.7 ± 2.11	51.4 ± 10.7	0.72
129	73.9 ± 13.2	62.1 ± 9.05	0.84
**Bkgd**	**Str and End Cell Count (cells/200 µm of cornea)**		
	Wt	Het	Het/Wt
B6	18.5 ± 1.98	29.0 ± 9.95	1.57
F1	20.8 ± 5.78	23.3 ± 7.99	1.12
129	16.2 ± 2.67	21.5 ± 2.87	1.33

Epithelium (Epi), *Pax6*$^{+/+}$ (Wt), *Pax6*$^{Sey/+}$ (Het), ratio of Het measurement to Wt measurement (Het/Wt), genetic background (bkgd), C57BL/6J (B6), B6129F1 (F1), 12921/SvImJ (129), stroma (Str), endothelium (End). Data is reported as mean ± standard deviation.

Table S5. Mean and relative Wt-, *Sey*-, and non-specific *Pax6* mRNA levels

Tissue	Bkgd	Wt-Specific mRNA (normalized transcripts)			
		Wt	Het	Hom	Het/Wt
**E18.5 Brain**	B6	0.33 ± 0.07	0.22 ± 0.02	0.00 ± 0.00	0.67
	F1	0.35 ± 0.10	0.22 ± 0.01	0.00 ± 0.00	0.63
	129	0.33 ± 0.04	0.24 ± 0.05	0.00 ± 0.00	0.73
**Adult Retina**	B6	1.19 ± 0.31	0.67 ± 0.37	-	0.56
	F1	1.78 ± 0.07	0.74 ± 0.16	-	0.42
	129	2.42 ± 0.49	1.05 ± 0.07	-	0.43
**Adult Cornea**	B6	3.26 ± 1.97	0.48 ± 0.20	-	0.15
	F1	4.42 ± 1.41	1.26 ± 0.63	-	0.29
	129	4.90 ± 0.79	1.80 ± 0.66	-	0.37
**Tissue**	**Bkgd**	**Sey-Specific mRNA (normalized transcripts)**			
		Wt	Het	Hom	Het/Wt
**E18.5 Brain**	B6	0.00 ± 0.00	0.06 ± 0.01	0.27 ± 0.03	-
	F1	0.00 ± 0.00	0.06 ± 0.02	0.30 ± 0.06	-
	129	0.00 ± 0.00	0.07 ± 0.01	0.31 ± 0.05	-
**Adult Retina**	B6	0.00 ± 0.00	0.14 ± 0.10	-	-
	F1	0.01 ± 0.02	0.28 ± 0.09	-	28.0
	129	0.01 ± 0.01	0.41 ± 0.06	-	41.0
**Adult Cornea**	B6	0.00 ± 0.00	0.13 ± 0.09	-	-
	F1	0.00 ± 0.00	0.20 ± 0.10	-	-
	129	0.00 ± 0.00	0.33 ± 0.17	-	-
**Tissue**	**Bkgd**	**Non-Specific mRNA (normalized transcripts)**			
		Wt	Het	Hom	Het/Wt
**E18.5 Brain**	B6	0.35 ± 0.04	0.40 ± 0.02	0.45 ± 0.08	1.14
	F1	0.46 ± 0.06	0.38 ± 0.04	0.54 ± 0.09	0.86
	129	0.46 ± 0.02	0.43 ± 0.10	0.55 ± 0.09	0.83
**Adult Retina**	B6	1.66 ± 0.39	0.99 ± 0.41	-	0.60
	F1	2.20 ± 0.05	1.14 ± 0.21	-	0.52
	129	2.53 ± 0.10	1.46 ± 0.10	-	0.58
**Adult Cornea**	B6	4.90 ± 1.13	1.28 ± 0.49	-	0.26
	F1	9.33 ± 3.66	2.45 ± 1.23	-	0.26
	129	10.8 ± 2.10	4.17 ± 1.09	-	0.39

Pax6$^{+/+}$ (Wt), *Pax6*$^{Sey/+}$ (Het), *Pax6*$^{Sey/Sey}$ (Hom), ratio of Het measurement to Wt measurement (Het/Wt), embryonic day 18.5 (E18.5), genetic background (bkgd), C57BL/6J (B6), B6129F1 (F1), 12921/SvImJ (129). Data is reported as mean ± standard deviation.

Table S6. Mean and relative PAX6 protein levels

Tissue	Bkgd	Protein (normalized arbitrary units)			
		Wt	Het	Hom	Het/Wt
**E18.5 Brain**	B6	17.8 ± 5.13	12.9 ± 1.94	1.19 ± 0.89	0.72
	F1	18.1 ± 4.05	10.6 ± 2.27	1.00 ± 0.60	0.58
	129	15.8 ± 3.94	10.3 ± 2.00	0.47 ± 0.69	0.65
**Adult Retina**	B6	86.5 ± 19.3	45.8 ± 16.2	-	0.53
	F1	114 ± 21.1	64.7 ± 18.4	-	0.57
	129	85.1 ± 20.0	62.5 ± 12.0	-	0.73
**Adult Cornea**	B6	428 ± 49.6	300 ± 103	-	0.70
	F1	410 ± 178	177 ± 17.6	-	0.43
	129	382 ± 111	226 ± 147	-	0.59

Pax6$^{+/+}$ (Wt), *Pax6*$^{Sey/+}$ (Het), *Pax6*$^{Sey/Sey}$ (Hom), ratio of Het measurement to Wt measurement (Het/Wt), embryonic day 18.5 (E18.5), genetic background (bkgd), C57BL/6J (B6), B6129F1 (F1), 12921/SvImJ (129). Data is reported as mean ± standard deviation.

Table S7. Mean and relative embryonic blood glucose levels

Bkgd	Blood Glucose (mmol/L)			
	Wt	Het	Hom	Het/Wt
B6	3.49 ± 0.20	3.94 ± 0.75	5.34 ± 0.94	1.13
F1	4.89 ± 1.36	4.26 ± 0.89	6.75 ± 1.71	0.87
129	2.68 ± 0.15	2.59 ± 0.38	2.52 ± 0.41	0.97

Pax6$^{+/+}$ (Wt), *Pax6*$^{Sey/+}$ (Het), *Pax6*$^{Sey/Sey}$ (Hom), ratio of Het measurement to Wt measurement (Het/Wt), genetic background (bkgd), C57BL/6J (B6), B6129F1 (F1), 12921/SvImJ (129). Data is reported as mean ± standard deviation.
